# Impact of pediatric obesity on abdominal surgery outcome: a narrative review

**DOI:** 10.3389/fped.2026.1780811

**Published:** 2026-03-25

**Authors:** Gloria Pelizzo, Valeria Calcaterra, Michela Marinaro, Eleonora Durante, Ilaria Anna Maria Scavone, Carlotta Paola Maria Canonica, Valentina De Giorgis, Gianvincenzo Zuccotti

**Affiliations:** 1 Department of Biomedical and Clinical Science, University of Milan, Milan, Italy; 2 Pediatric Surgery Department, Buzzi Children’s Hospital, Milan, Italy; 3 Pediatrics and Adolescentology Unit, Department of Internal Medicine, University of Pavia, Pavia, Italy; 4 Pediatric Department, Buzzi Children’s Hospital, Milan, Italy; 5 Anesthesia and Intensive Care Unit, Vittore Buzzi Children’s Hospital, Milan, Italy

**Keywords:** adolescents, children, obesity, overweight, pediatric surgery, surgery complications

## Abstract

**Background:**

Childhood obesity has become a major global health concern and represents an increasingly relevant modifier of perioperative risk. In pediatric abdominal surgery, obesity is associated with anatomical, physiological, and immunometabolic alterations that may influence intraoperative management and postoperative outcomes. This narrative review aims to synthesize current evidence on the impact of obesity on surgical outcome in children undergoing abdominal procedures.

**Methods:**

A narrative literature review was conducted using PubMed, Scopus, and Embase, focusing on studies published in the last 15 years. Evidence was qualitatively synthesized and organized thematically, prioritizing large cohort studies and national registries. No formal risk-of-bias assessment or meta-analysis was performed.

**Results:**

Available evidence indicates that children with obesity undergoing abdominal surgery experience higher rates of perioperative respiratory adverse events, wound complications, prolonged operative times, and longer hospital stays, particularly in the presence of comorbidities such as obstructive sleep apnea syndrome (OSAS) and cardiometabolic dysfunction. The impact of obesity varies by procedure, with heterogeneous outcomes reported across appendectomy, colorectal surgery, and other abdominal interventions. Multidisciplinary perioperative strategies, including optimized airway management, multimodal opioid-sparing analgesia, respiratory physiotherapy, and thromboembolic prophylaxis, are associated with improved outcomes.

**Conclusions:**

Obesity should be regarded as a risk amplifier rather than a contraindication to pediatric abdominal surgery. Tailored perioperative management and the adoption of obesity-adapted Enhanced Recovery After Surgery pathways may reduce complications, optimize resource utilization, and improve outcomes in this growing pediatric population.

## 1 Introduction

Over the past decades, childhood obesity has evolved from a relatively uncommon condition to a major global public health challenge. Large population-based analyses have demonstrated a dramatic increase in the prevalence of overweight and obesity among children and adolescents worldwide, rising from less than 1% in 1975 to over 7% in boys and 5%–6% in girls by 2016 (1). Recent global estimates indicate that more than 38 million children under the age of five are affected by overweight or obesity, with prevalence rates exceeding 20%–30% in many high-income countries and rapidly increasing in low- and middle-income regions (2–5).

This epidemiological trend is increasingly reflected in surgical practice, where a growing number of pediatric patients with obesity undergo elective and emergency surgical procedures. Recent studies of pediatric surgical cohorts report that overweight and obesity may affect approximately 15%–20% of children undergoing surgical procedures, indicating that the prevalence in surgical populations may be even higher than in the general pediatric population (6, 7). Obesity is now recognized as an independent perioperative risk factor in children, associated with higher rates of respiratory, cardiovascular, metabolic, and wound-related complications (8–10). In abdominal surgery, excess subcutaneous and visceral adipose tissue contributes to increased technical difficulty, prolonged operative times, and greater physiological stress, even in minimally invasive procedures (9, 11–14).

Beyond mechanical factors, obesity is also characterized by chronic low-grade systemic inflammation, which contributes to endothelial dysfunction, insulin resistance, and altered responses to surgical stress (15–20).

These immunometabolic alterations may contribute to impaired wound healing and increased susceptibility to postoperative complications (18, 21–24).

These mechanisms are particularly relevant in pediatric abdominal surgery, where surgical stress and anesthetic management interact with the altered cardiopulmonary and metabolic physiology of children with obesity (25, 26). Several pediatric cohort studies have reported higher rates of perioperative respiratory events, wound complications, and prolonged hospital stay in children with obesity (6, 12, 22).

Despite the growing body of literature addressing obesity-related risks in pediatric surgery, existing evidence remains heterogeneous and often procedure-specific, with limited integration of epidemiological trends, pathophysiological mechanisms, and clinical outcomes. In particular, the role of obesity-associated inflammation as a unifying pathway linking excess adiposity to surgical morbidity in children has not been systematically explored.

Therefore, this narrative review aims to synthesize current evidence on complications of abdominal surgery in children with obesity, with a specific focus on the interplay between epidemiological burden, obesity-related inflammation, and perioperative surgical outcomes. By integrating anatomical, pathophysiological, and clinical perspectives, this review seeks to provide a comprehensive framework for risk stratification and perioperative management in this increasingly prevalent and vulnerable pediatric population. In addition, this review aims to highlight current knowledge gaps and future research priorities in the perioperative management of pediatric patients with obesity.

## 2 Methods

This study was conducted as a narrative review aimed at summarizing and critically discussing the available evidence on perioperative and postoperative complications of abdominal surgery in children with obesity. Given the heterogeneity of study designs, surgical procedures, and outcome measures in this field, a narrative approach was considered appropriate to integrate epidemiological data, pathophysiological mechanisms, and clinical outcomes without the constraints of a systematic review methodology.

A comprehensive literature search was performed using the electronic databases PubMed, Embase, and Scopus, focusing on studies published between December 2010 and November 2025 (last 15 years) in order to reflect contemporary surgical techniques, anesthetic practices, and perioperative care pathways. The search strategy combined Medical Subject Headings (MeSH) and free-text terms related to pediatric obesity and surgery. The main search terms included: “*obesity*”, “*pediatric obesity*”, “*child*”, “*adolescent*”, “*abdominal surgery*”, “*general surgery*”, “*perioperative complications*”, “*postoperative complications*”, “*anesthesia*”, “*wound healing*”, “*obstructive sleep apnea syndrome*”, “*cardiovascular disease*”, and “*inflammation*”. Boolean operators (AND, OR) were used to combine the terms, for example: *(obesity OR pediatric obesity) AND (child OR adolescent) AND (abdominal surgery OR general surgery) AND (perioperative complications OR postoperative complications)*. The complete database-specific search strings are reported in Supplementary Table S1.

Eligible studies included observational studies, retrospective and prospective cohort studies, registry-based analyses, clinical trials, systematic reviews, and meta-analyses focusing on children and adolescents with obesity undergoing abdominal surgical procedures. Studies exclusively focused on bariatric surgery, or not reporting surgical or anesthesiologic outcomes, were excluded. Studies conducted in adult populations were considered only when they provided relevant insights into general pathophysiological mechanisms applicable to pediatric obesity and perioperative risk.

The initial search retrieved 980 records. The initial search retrieved 980 records. Duplicate records identified across databases were removed using Rayyan prior to the title and abstract screening phase. Titles and abstracts were screened for relevance by two authors, and studies that did not meet the inclusion criteria (adult-only populations, absence of surgical relevance, or lack of perioperative or postoperative outcome data) were excluded (*n* = 823). The full texts of potentially relevant articles were subsequently assessed to determine eligibility for inclusion in the narrative synthesis. Any uncertainties regarding study inclusion were resolved through discussion among the authors until consensus was reached.

**Figure 1:**
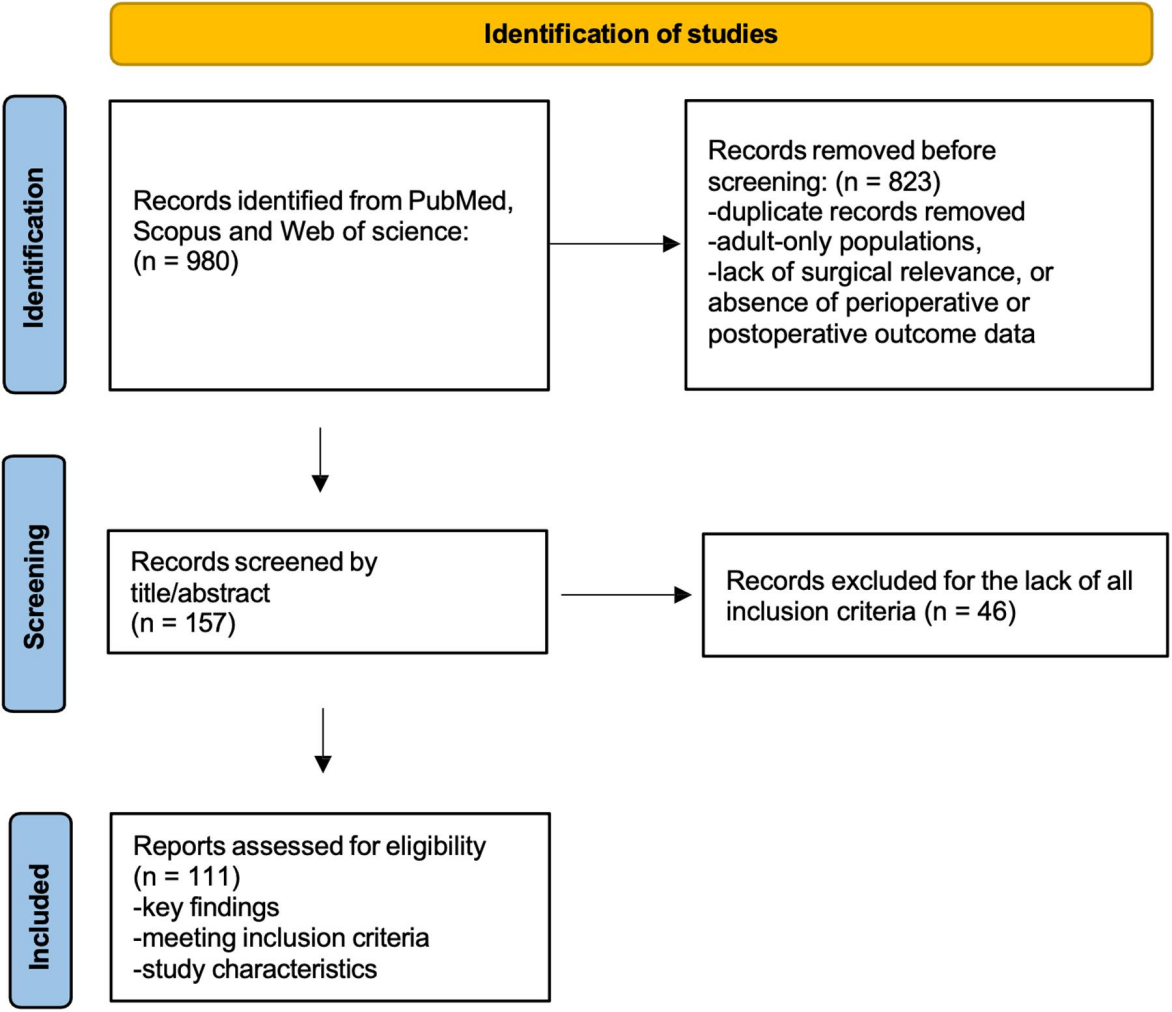
Flow diagram of the study selection process for the review.

After screening, 157 articles underwent full-text assessment, and 111 studies were ultimately included in the narrative synthesis based on their relevance to the objectives of the review. Reference lists of the included studies were also manually screened to identify additional relevant publications. A simplified flow diagram summarizing the study selection process has been included to improve transparency (Figure 1).

Data from the included studies were synthesized qualitatively and thematically, focusing on perioperative risk factors, anesthetic considerations, surgical outcomes, and postoperative complications in pediatric patients with obesity. Particular attention was paid to study design and data sources, distinguishing between prospective and retrospective cohort studies, registry-based analyses, and large administrative datasets. Although a formal risk-of-bias assessment was not performed due to the narrative nature of the review and the heterogeneity of the included studies, a qualitative appraisal of study characteristics and methodological limitations was considered during interpretation of the evidence. In particular, findings derived from large databases (such as NSQIP-Pediatric and similar national registries) were interpreted in light of their inherent limitations, including potential coding inaccuracies, limited granularity of clinical variables, and restricted availability of long-term outcomes. Areas of heterogeneity or conflicting evidence across studies were identified and discussed in the relevant sections of the manuscript.

## 3 Pathophysiological and surgical implications of obesity

Understanding the pathophysiological features of obesity in childhood is essential to correctly interpret surgical risk and perioperative vulnerability in pediatric patients (27). Obesity is not merely a condition of excess body weight, but a complex systemic disease characterized by anatomical, metabolic, and immunological alterations that significantly modify the response to surgical stress (28, 29).

From an anatomical perspective, excess adipose tissue, particularly visceral fat, alters body composition and organ relationships (30). During abdominal surgery, increased thickness of the abdominal wall and accumulation of intra-abdominal fat may limit surgical exposure, complicate identification of anatomical planes, and increase operative complexity (31). These features have been associated with longer operative times and, in selected cases, higher conversion rates from minimally invasive to open surgery, even in pediatric populations (32–34).

Beyond mechanical factors, pediatric obesity is associated with relevant respiratory and cardiovascular physiological changes (35). Excess of thoracoabdominal fat reduces chest wall compliance, functional residual capacity, and ventilatory reserve, predisposing children with obesity to hypoventilation and impaired gas exchange during anesthesia (36–38). Central fat distribution, particularly abdominal and cervical adiposity, is also associated with narrowing of the upper airways and a higher prevalence of sleep-disordered breathing, including obstructive sleep apnea syndrome (OSAS) (35, 39–43). Similarly, early cardiovascular changes, including increased blood volume, elevated cardiac output, and increased left ventricular mass, create a hyperdynamic circulatory state that may reduce physiological reserve during surgical stress (8, 9, 44, 45).

A central and unifying mechanism linking obesity to adverse surgical outcomes is the presence of chronic low-grade systemic inflammation. Visceral adipose tissue acts as an active endocrine and immunological organ, producing pro-inflammatory cytokines such as tumor necrosis factor-α (TNF-α), interleukin-6 (IL-6), and monocyte chemoattractant protein-1 (MCP-1) (16, 17, 46). This inflammatory milieu promotes endothelial dysfunction, insulin resistance, oxidative stress, and dysregulated immune responses, resulting in an exaggerated reaction to surgical trauma and impaired resolution of inflammation (15, 18–20).

These immunometabolic alterations have direct implications into wound healing and postoperative recovery. Chronic inflammation, combined with relative tissue hypoperfusion and altered macrophage and fibroblast activity, interferes with angiogenesis, collagen deposition, and tissue remodeling (47). Consequently, children with obesity are biologically predisposed to wound-related complications following abdominal surgery (21–24).

Obesity also affects vascular homeostasis and coagulation (48). Although venous thromboembolism remains uncommon in the pediatric population, obesity contributes to a prothrombotic state through endothelial activation, inflammatory mediator release, and impaired fibrinolysis (49). This risk becomes clinically relevant in the presence of additional factors such as surgical trauma, prolonged immobilization, or adolescence and post-puberal state, particularly after major abdominal procedures (22, 50–52).

Importantly, many of these pathophysiological alterations are dynamic rather than irreversible. Evidence from pediatric and adolescent populations shows that weight reduction, achieved through lifestyle interventions or bariatric surgery, is associated with a significant reduction in systemic inflammation and partial restoration of endothelial and metabolic function (8, 53, 54). These observations provide the biological rationale for preoperative optimization strategies and tailored perioperative pathways for elective surgery.

Pediatric obesity induces a constellation of anatomical, physiological, and immunometabolic changes that collectively reduce surgical resilience and amplify the response to operative stress (55). Taken together, these mechanisms help explain the increased perioperative vulnerability observed in children with obesity undergoing abdominal surgery.

Table 1 outlines the main pathophysiological features of obesity in children that are relevant to perioperative management.

**Table 1:** Perioperative optimization strategies in children with obesity and enhanced recovery after surgery (ERAS)-based management (56–58).

Condition in pediatric patients with obesity	System abnormality	Clinical risk	ERAS-based management
Metabolic abnormalities	Increased risk of insulin resistance and diabetes	Hyperglycemia, infection, impaired wound healing	- Glucose monitoring; - Avoid prolonged fasting; - Carbohydrate loading when appropriate
Adipose tissue	Augmented inflammatory status and response	Increased surgical stress, infection risk	- Preferred minimally invasive surgery; - Nutritional optimization;
Drug metabolism	Altered pharmacokinetics and pharmacodynamics	Risk of under- or overdosing	- Dosing based on lean body mass calculations and allometric scaling models; - Opioid-sparing strategies
Cardiopulmonary reserve	Reduced lung compliance and increased work of breathing	Hypoxemia, atelectasis, respiratory complications	- Preoperative assessment; - Lung-protective ventilation; - Early mobilization
Airway management	Potentially difficult airway	Difficult intubation	- Anticipatory airway planning; - Difficult airway equipment
Mobility	Reduced functional mobility	Thromboembolism, delayed recovery	- Early mobilization; - Thromboprophylaxis when indicated
Nutrition	Nutritional imbalance despite obesity	Postoperative catabolism	- Early oral feeding; individualized nutritional support
Postoperative pain	Increased analgesic requirements	Respiratory depression	- Multimodal analgesia; - Regional anesthesia when feasible

The content represents an evidence-informed author synthesis and should not be interpreted as formal clinical guideline recommendations.

## 4 Surgical and anesthesiological management

Pediatric obesity, whose prevalence has increased dramatically in recent decades, represents a significant risk factor for intraoperative surgical and anesthesiologic complications (26, 34).

### 4.1 Anesthesiological management (pharmacological and airway management)

Obesity significantly alters drug pharmacokinetics and pharmacodynamics by altering body composition, volume of distribution, protein binding, hepatic metabolism, and renal clearance (10, 59). These alterations complicate anesthetic dosing, particularly for intravenous agents, opioids, and sedatives (2, 4, 60).

Dosing based on total body weight may lead to overdosing of lipophilic drugs, whereas dosing based on ideal body weight may result in underdosing of hydrophilic agents. Consequently, lean body mass-based calculations and allometric scaling models are often more appropriate for anesthetic dosing in pediatric patients with obesity (50, 51, 61). These pharmacological considerations require careful intraoperative titration and close monitoring to avoid delayed emergence, respiratory depression, or inadequate anesthesia.

Airway management in children with obesity is frequently challenging because of altered anatomy, including cervical adiposity, relative macroglossia, reduced neck mobility, and narrowing of the posterior pharyngeal space (9, 62). These anatomical features are commonly associated with OSAS, which is highly prevalent in pediatric patients with obesity and represents an important predictor of perioperative respiratory adverse events (39–43).

Children with obesity also exhibit reduced pulmonary compliance, decreased functional residual capacity, and increased oxygen consumption, resulting in limited apnea tolerance, particularly in the supine position and under general anesthesia (27, 63, 64). Large observational studies, including the PEACHY study, have demonstrated higher rates of difficult mask ventilation, difficult laryngoscopy, intraoperative desaturation, and postoperative airway obstruction in children with obesity compared with normal-weight peers (9, 65).

For these reasons, protective ventilation strategies (such as low tidal volumes, appropriate positive end-expiratory pressure (PEEP), and recruitment maneuvers) are recommended (66). Multimodal analgesia is also encouraged to reduce opioid requirements and minimize the risk of postoperative respiratory depression (9).

### 4.2 Surgical technical challenges, intra- and post-operative complications

Pediatric obesity is widely recognized as an independent predictor of postoperative complications following abdominal surgery (67, 68). Multiple cohort studies and national databases have consistently demonstrated higher rates of morbidity, prolonged postoperative recovery, and increased healthcare utilization in children with obesity compared with their normal-weight peers (22, 67, 69).

Children with obesity exhibit substantial anatomical and physiological alterations involving multiple organ systems. Tissue perfusion and angiogenesis are impaired in this population, negatively affecting wound healing and the response to surgical stress.

From an intraoperative standpoint, children with obesity are at increased risk of pressure necrosis, peripheral nerve injury, and rhabdomyolysis, making meticulous patient positioning on the operating table and accurate padding essential (64, 70).

As a result of the chronic inflammatory state associated with obesity, the risk of wound dehiscence, seroma formation, and surgical site infection increases proportionally with body mass index (BMI) (12, 21, 23, 71). Data from the National Surgical Quality Improvement Program–Pediatric (NSQIP-Pediatric) database confirm pediatric obesity as an independent predictor of wound complications during and after abdominal and thoracic surgery (22). Given this tissue fragility, large multicenter studies suggest that minimally invasive abdominal surgery, when performed in optimized settings, should be considered the treatment of choice in pediatric patients with obesity.

Laparoscopic appendectomy, the most common abdominal procedure in children with obesity, may be associated with an increased risk of vascular injury and hematoma during trocar placement. Although laparoscopy offers important advantages, it can present technical challenges due to increased abdominal wall thickness and excess adipose tissue, which may limit transillumination, operative exposure, and instrument maneuverability. Therefore, these procedures are best performed in centers experienced in the management of pediatric patients with obesity (9, 11, 14, 72). Despite the longer operative times compared to open approaches, minimally invasive surgery does not appear to be associated with significantly higher rates of major intraoperative complications or intra-abdominal abscess formation (73).

Postoperative respiratory complications, including hypoxemia, atelectasis, pneumonia, and airway obstruction, occur more frequently in children with obesity, particularly in those with pre-existing asthma or OSAS (6, 37). Increased subdiaphragmatic fat deposition and reduced lung compliance predispose patients with obesity to postoperative pulmonary complications, even after minimally invasive surgery (12, 38).

Systemic complications such as sepsis and metabolic instability have also been reported (74, 75), reflecting the interaction between surgical stress and obesity-related immunometabolic dysregulation (76).

Obesity has been associated with increased length of hospital stay (LOS), higher rates of readmission, and greater likelihood of unplanned hospital admission following outpatient surgery (67, 69, 77, 78). These findings have important implications for fast-track and ambulatory surgical pathways, suggesting that children with obesity may require individualized postoperative monitoring and dedicated discharge planning.

Overall, the available evidence indicates that pediatric obesity increases perioperative complexity and the risk of specific complications, highlighting the need for tailored anesthesiologic management, careful surgical planning, and multidisciplinary perioperative care.

## 5 Procedure-specific outcomes in pediatric abdominal surgery

The impact of obesity on surgical outcomes in children varies according to the type of abdominal procedure performed, underscoring the need for procedure-specific risk stratification and individualized perioperative management (7, 79).

Appendectomy is the most frequently performed abdominal procedure in pediatric patients with obesity. Several studies have reported higher rates of complicated or perforated appendicitis in children with obesity, with perforation rates reported in approximately 30%–50% of cases compared with 20%–30% in normal-weight peers, often attributed to delayed diagnosis and atypical clinical presentation (80–82). Obesity has also been associated with longer operative times and an increased incidence of postoperative intra-abdominal abscesses (7, 12, 68, 77, 81). However, when minimally invasive or single-port appendectomy techniques are employed, many authors have not observed a significant increase in postoperative complications compared with normal-weight peers (83–85). These findings suggest that surgical approach and institutional experience may play a more important role than obesity alone in determining outcomes.

In pediatric colorectal surgery, obesity has been associated with higher rates of deep SSI and failure of minimally invasive surgery in selected cohorts (69, 79). Conversely, other large studies have not identified BMI as an independent predictor of postoperative morbidity, highlighting the heterogeneity of available evidence (67).

In procedures such as cholecystectomy, renal transplantation, and continent urinary tract reconstruction, obesity has not consistently been associated with increased postoperative complications, readmission rates, or length of hospital stay (76, 86–88). These findings support a nuanced, procedure-specific interpretation of obesity-related surgical risk.

Table 2 summarizes the main studies reporting perioperative and postoperative outcomes of abdominal surgery in children with obesity.

**Table 2:** Summary of studies reporting outcomes of abdominal surgery in children with obesity.

Author (year)	Number of patients	Type of surgery	Main reported complications/outcomes
Blanco et al. (81)	1,078	Appendectomy	Higher incidence of perforated appendicitis in children with obesity
Garey et al. (76)	1,258	Appendectomy (perforated)	Longer operative time, increased length of stay, higher rate of postoperative abscess
Witt et al. (70)	3,868	Gastrointestinal surgery	Higher rates of superficial SSI, unplanned intubation, postoperative seizures
Michailidou et al. (12)	2,312	Laparoscopic appendectomy	Increased rate of complicated appendicitis, deep SSI, longer operative time
Petnehazy et al. (85)	180	Single-port appendectomy	No significant differences in postoperative complications compared with normal-weight patients
Papillon et al. (84)	1,509	Appendectomy	No differences in postoperative complications, LOS, abscess formation, or readmission
Hebballi et al. (83)	4,160	Appendectomy	No association with complicated appendicitis; higher rate of postoperative complications
Kao et al. (71)	707	Colorectal surgery (IBD)	Higher risk of deep surgical site infection in patients with obesity
Garey et al. (76)	356	Cholecystectomy	No difference in LOS or postoperative complication rate
Richter et al. (87)	456	Kidney transplantation	No significant difference in early postoperative complications
Strine et al. (86)	350	Continent urinary tract reconstruction	No difference in postoperative complications or readmission rates

Studies included in this table represent a selected subset of the studies identified in the narrative synthesis and were included because they reported procedure-specific surgical outcomes in pediatric patients with obesity.

LOS, length of stay; SSI, surgical site infection.

Taken together, these findings indicate that the impact of obesity on surgical outcomes in children is heterogeneous and largely procedure-dependent, reinforcing the importance of individualized perioperative assessment and experienced surgical management.

## 6 Perioperative optimization and prevention strategies

Optimal perioperative management is crucial for improving surgical outcomes and reducing complications in children with obesity undergoing abdominal surgery. Given the multifactorial nature of obesity-related risk, preventive and optimization strategies must be implemented across all phases of care, from preoperative assessment to postoperative recovery. A structured, multidisciplinary approach is essential to address modifiable risk factors and to tailor perioperative pathways to the specific physiological vulnerabilities of this population.

In children and adolescents with obesity, preoperative care requires a structured and comprehensive approach beginning with the objective identification of obesity using BMI and BMI-for-age percentiles. The perioperative setting represents a valuable opportunity for early recognition of pediatric obesity and associated comorbidities, which are frequently underdiagnosed despite their clinical relevance (89).

A complete preoperative assessment should include systematic screening for obesity-related comorbidities, such as OSAS, asthma, hypertension, insulin resistance or type 2 diabetes, dyslipidemia, and nonalcoholic fatty liver disease (45, 89). Particular attention should be given to respiratory symptoms, sleep-disordered breathing, baseline oxygen saturation, and exercise tolerance, as these factors are strongly associated with perioperative respiratory adverse events.

Airway assessment is a critical component of preoperative evaluation and should include direct examination of airway anatomy, assessment of neck and jaw mobility, mouth opening, and identification of predictors of difficult mask ventilation or tracheal intubation. Early identification of high-risk patients allows appropriate anesthetic planning, selection of airway devices, and postoperative monitoring strategies.

Although routine extensive laboratory or cardiac investigations are not recommended for all children with obesity, targeted evaluation is warranted when clinical history or examination suggests increased cardiometabolic risk. In selected high-risk cases, preoperative optimization may involve referral to a multidisciplinary team. Preventive strategies aimed at reducing infectious and thromboembolic complications are also central to perioperative management. While antibiotic prophylaxis practices do not substantially differ across BMI categories, obesity is consistently associated with a higher risk of surgical site infection (SSI), highlighting the importance of strict adherence to prophylactic protocols (90–92). Antibiotic prophylaxis should be administered within the recommended time window before surgical incision to ensure adequate tissue concentrations, with careful consideration of dosing in relation to body weight and altered pharmacokinetics in obesity (93). Although specific pediatric dosing guidelines tailored to obesity are limited, attention to appropriate timing, intraoperative redosing during prolonged procedures, and meticulous skin and wound care remains essential to minimize infectious risk (58, 93).

Venous thromboembolism (VTE), while rare in the pediatric population, is increasingly recognized in adolescents with obesity, particularly in the presence of additional risk factors such as major abdominal surgery, prolonged immobilization, central venous catheters, or severe OSAS (50, 51, 61). Current evidence does not support routine pharmacologic thromboprophylaxis in all children with obesity; instead, an individualized risk assessment is recommended (94).

In selected high-risk patients, pharmacologic prophylaxis with low-molecular-weight heparin may be considered, in combination with mechanical measures such as sequential compression devices, to reduce venous stasis and thrombotic risk (90). When used, dosing should be weight-based and adjusted for obesity-related pharmacokinetic changes, with careful monitoring for bleeding complications (94–97).

These considerations underscore the importance of structured multidisciplinary perioperative pathways aimed at early risk identification, individualized anesthetic and surgical planning, and prevention of respiratory, infectious, and thromboembolic complications in pediatric patients with obesity.

## 7 Postoperative recovery strategies

Postoperative management represents a critical phase for preventing complications in children with obesity, who are particularly vulnerable to respiratory compromise, venous stasis, and delayed functional recovery (98, 99). Early mobilization is fundamental during postoperative care, as it reduces the risk of pulmonary complications and thromboembolic events while promoting gastrointestinal recovery and overall functional restoration (100).

Pain management should prioritize multimodal, opioid-sparing strategies to minimize the risk of respiratory depression, especially in children with OSA or reduced respiratory reserve (101, 102). Effective analgesia combines regional anesthesia techniques, non-opioid systemic analgesics, Non-Steroidal Anti-Inflammatory Drugs (NSAIDs), and acetaminophen, with carefully titrated opioids when necessary (101, 103–105). Employing multiple agents and techniques with complementary mechanisms of action, such as anti-inflammatory effects, modulation of neuropathic pain, and attenuation of central sensitization, provides more robust and comprehensive analgesia within a multimodal regimen (101, 103, 106).

Regional anesthesia methods provide a powerful opioid-sparing effect. Techniques such as epidural analgesia, transversus abdominis plane blocks, and rectus sheath blocks offer segmental anesthesia lasting 12–24 h after surgery, reducing overall opioid requirements by approximately 30%–50% while preserving respiratory function (104).

Respiratory physiotherapy plays a vital role in the care of children with obesity, who face an increased risk of postoperative pneumonia and atelectasis due to impaired baseline lung mechanics, reduced functional residual capacity, perioperative airway edema, and prolonged anesthetic-induced respiratory depression (100, 107).

Although specific postoperative respiratory rehabilitation protocols tailored exclusively to children with obesity are not consistently described in the literature, preventive respiratory physiotherapy has been shown to reduce postoperative pulmonary complications through several mechanisms (108). These include improving lung expansion and ventilation, facilitating clearance of airway secretions to prevent obstruction-related atelectasis, and maintaining chest wall mobility, even in the presence of postoperative pain (100, 108).

Implementing prophylactic respiratory physiotherapy, particularly when combined with effective pain control that preserves adequate respiratory effort, can reduce major postoperative respiratory complications and hospital-acquired pneumonia by approximately 45% (108). Recent evidence also suggests that lung recruitment strategies, such as early use of high-flow nasal cannula or appropriately applied noninvasive positive pressure ventilation, may further decrease the incidence of atelectasis in pediatric patients with obesity (109).

The intensity of respiratory support should be guided by individualized risk assessment, with more aggressive interventions reserved for children with sleep-disordered breathing, severe baseline hypoxemia, or neuromuscular comorbidities (99). These respiratory physiotherapy protocols and mobilization milestones are cost-effective, low-risk interventions that can be implemented across a wide range of healthcare settings with standard nursing and physiotherapy resources (110).

Enhanced Recovery After Surgery (ERAS) pathways provide a comprehensive framework for integrating perioperative optimization strategies in pediatric patients with obesity. By coordinating anesthetic, surgical, nursing, and rehabilitative care within structured protocols, this approach addresses key obesity-related challenges, including altered drug metabolism, reduced cardiopulmonary reserve, and increased inflammatory response (56, 57).

Adaptation of ERAS principles to pediatric obesity includes standardized preoperative assessment, optimized analgesia, early feeding, early mobilization, and structured postoperative monitoring (111, 112). Because children with obesity frequently present with metabolic abnormalities or an increased risk of diabetes (55, 113–115), perioperative glucose monitoring, using point-of-care testing or continuous glucose monitoring when available, and individualized insulin regimens are recommended to maintain blood glucose between 140 and 180 mg/dL during and immediately after surgery (55, 116).

Prevention of postoperative nausea and vomiting (PONV) through multimodal strategies represents another key ERAS component (117), as children with obesity have a higher baseline risk of PONV due to increased gastroesophageal reflux, delayed gastric emptying, and altered drug metabolism (58). Perioperative nutrition and ileus prevention are also integral to ERAS pathways, supporting gastrointestinal recovery and reducing postoperative ileus through coordinated nutritional and mobilization strategies (58).

Overall, this approach shift perioperative care from isolated interventions to an integrated, patient-centered approach, making them particularly well-suited to the complex and multidimensional challenges posed by pediatric obesity and central to the prevention of surgical complications in this growing population (57).

Tables 3, 4 summarize key perioperative considerations in children with obesity undergoing abdominal surgery.

**Table 3:** Pre and post-operative strategies in children with obesity undergoing abdominal surgery.

Phase	Domain	Strategies/Key points
Preoperative care	Antibiotic prophylaxis	Administer within 60 min before incision. Clean/clean-contaminated surgery: 1st line antibiotic is Cefazolin 30 mg/kg IV (max 2–3 g) (118). Contaminated surgery: Cefazolin + Metronidazole or Cefoxitin (if β-lactam allergy: Clindamycin + Gentamicin or Vancomycin + Gentamicin).
	Nursing care	Assessment of obstructive sleep apnea (OSA), asthma, and metabolic risk; Planned venous access; Patient and caregiver education; Anxiety reduction.
	Nutritional optimization	Avoid prolonged fasting; clear fluids until 2 h preoperatively; Consider carbohydrate loading in selected elective cases.
Intraoperative care	Patient positioning	Supine position with attention to abdominal compression; Reverse Trendelenburg if needed; Protection of pressure points.
	Anesthesiologic treatment	Opioid-sparing multimodal anesthesia: paracetamol (acetaminophen) + Non-Steroidal Anti-Inflammatory Drugs; regional anesthesia (e.g., TAP block); cautious opioid use; lung-protective ventilation.
Surgical care	Surgical approach	Minimally invasive (laparoscopic) approach preferred: reducing tissue trauma and shorter length of stay.
	Tissue handling	Avoid large dissections; meticulous hemostasis; Avoid routine drains and nasogastric tubes.
Postoperative care	Nursing & respiratory monitoring	Enhanced monitoring for apnea and hypoventilation, especially in patients with OSA; Continuous pulse oximetry when indicated.
	Pain management	Continuation of multimodal, opioid-sparing analgesia; Consider prolonged regional techniques when available.
	Glycemic monitoring	Postoperative blood glucose monitoring, especially after major or complicated abdominal surgery.
	PONV prevention	Risk-stratified prophylaxis with ondansetron (0.1 mg/kg IV) ± dexamethasone (0.1 mg/kg IV) (119); Adequate hydration; minimization of opioid use.
	Feeding & rehabilitation	Early oral feeding within 24 h; Avoidance of routine nasogastric decompression; Early mobilization and respiratory physiotherapy.

The content represents an evidence-informed author synthesis and should not be interpreted as formal clinical guideline recommendations.

IV, intravenous; OSA, obstructive sleep apnea; TAP, transversus abdominis plane.

**Table 4:** Risks of surgical complications and preventive enhanced recovery after surgery (ERAS)- strategies.

Complication	Pathophysiological basis in pediatric obesity	Clinical impact/Severity	Preventive ERAS strategies
Wound dehiscence	Increased subcutaneous fat, impaired collagen synthesis, chronic inflammation	Delayed healing, re-intervention, prolonged hospitalization	Minimally invasive surgery; meticulous fascial closure; tension-free sutures; glycemic control
Incisional hernia (laparocele)	Elevated intra-abdominal pressure, impaired fascial healing	Late complication requiring surgical repair	Careful fascial closure; avoidance of excessive strain; early mobilization with abdominal support
Surgical site infection (SSI)	Reduced tissue perfusion, altered immune response, dead space	Frequent complication; increased LOS and readmissions	Weight-adjusted antibiotic prophylaxis (1st line Cefazolin); normothermia; glycemic control; minimally invasive approach
Seroma/wound hematoma	Excess adipose tissue and dead space formation	Predisposes to infection and wound breakdown	Careful hemostasis; limited dissection; selective use of drains
Bleeding related to trocar positioning	Thick abdominal wall, difficult landmarks	Vascular injury, conversion to open surgery	Ultrasound-guided trocar placement; careful port positioning; experienced laparoscopic technique
Port-site hernia (laparoscopy)	Increased abdominal wall thickness and pressure	Risk of bowel incarceration	Systematic fascial closure of ports ≥10 mm; appropriate port size
Intra-abdominal abscess	Higher SSI rates, impaired immune response	Sepsis risk, prolonged antibiotics	Adequate source control; minimally invasive drainage; early mobilization and feeding
Anastomotic leak (intestinal surgery)	Impaired tissue oxygenation and perfusion	High morbidity; Intensive Care Unit (ICU) admission	Careful patient selection; optimized tissue perfusion; avoidance of hypotension; early leak detection
Postoperative hypoxemia	Reduced lung compliance, obesity hypoventilation	Increased monitoring and ICU need	Lung-protective ventilation; early mobilization; incentive spirometry
Atelectasis	Pain, splinting, reduced mobility	Delayed recovery, respiratory failure	Multimodal opioid-sparing analgesia; respiratory physiotherapy; early ambulation
Airway edema	Difficult airway management, fluid overload	Post-extubation obstruction	Judicious fluid management; gentle airway manipulation; extubation readiness assessment
Aspiration pneumonia	Delayed gastric emptying, PONV	Severe pulmonary complication	PONV prophylaxis; avoidance of prolonged fasting; early feeding protocols
Venous thromboembolism (VTE)	Pro-thrombotic inflammatory state, immobility	Rare but potentially life-threatening	Early mobilization; mechanical thromboprophylaxis; pharmacologic prophylaxis in high-risk adolescents
Delayed gastric emptying/ileus	Autonomic dysfunction, opioid exposure	Delayed feeding and discharge	Opioid-sparing analgesia; early oral intake; avoidance of routine nasogastric tubes
Perioperative hyperglycemia	Insulin resistance, stress response	Infection risk, poor wound healing	Perioperative glucose monitoring; avoidance of prolonged fasting; ERAS nutritional protocols
Opioid-related respiratory depression	OSA, altered pharmacokinetics	Major safety concern	Multimodal analgesia; regional techniques; cautious opioid dosing
Delayed mobilization and recovery	Reduced baseline mobility, pain	Secondary complications, longer LOS	Structured early mobilization protocols; physiotherapy involvement

Data are presented as an evidence-informed author synthesis rather than formal guideline recommendations.

These strategies emphasize that postoperative recovery in children with obesity requires coordinated multidisciplinary care, combining early mobilization, optimized analgesia, respiratory physiotherapy, and ERAS-based protocols to reduce complications and support functional recovery.

Despite the growing body of literature on pediatric obesity and surgical outcomes, several important knowledge gaps remain. Much of the available evidence is derived from retrospective analyses and administrative databases, while prospective studies specifically designed to evaluate perioperative risk in children with obesity remain limited. In addition, variability in BMI definitions and obesity classifications across studies complicates comparison of outcomes and highlights the need for standardized criteria. Pediatric-specific perioperative management protocols tailored to obesity are still inconsistently described in the literature, and data on long-term postoperative outcomes and patient-centered measures remain scarce. Future research should prioritize prospective multicenter studies, standardized definitions of obesity, and the development of evidence-based perioperative pathways specifically designed for pediatric patients with obesity.

## 8 Limitations

This narrative review has several limitations that should be acknowledged when interpreting its findings. First, due to the narrative design, the literature search and study selection were not conducted according to a predefined systematic protocol, and no formal risk-of-bias assessment was performed. As a result, the review may be subject to selection bias and may not capture all relevant publications on the topic.

Second, the available evidence on surgical outcomes in pediatric patients with obesity is highly heterogeneous. Included studies differ in design, patient populations, definitions of obesity, surgical procedures, outcome measures, and follow-up duration. This heterogeneity limits direct comparison across studies and precludes quantitative synthesis or meta-analysis.

Third, much of the existing literature is retrospective and observational, often based on administrative databases or registries. While these sources provide large sample sizes and real-world data, they may be affected by residual confounding, incomplete clinical detail, and variability in reporting perioperative complications. Large datasets such as NSQIP-Pediatric provide valuable population-level insights but may be limited by coding variability, limited clinical granularity, and lack of long-term patient-centered outcomes.

In addition, obesity-related risk is frequently analyzed as a binary variable, without accounting for differences in obesity severity, body fat distribution, pubertal status, or metabolic phenotype, which may differentially influence surgical risk. Data on long-term outcomes and patient-centered measures, such as quality of life and functional recovery, are also limited.

Finally, although this review integrates epidemiological, pathophysiological, and clinical perspectives, the lack of standardized perioperative protocols specific to pediatric patients with obesity across studies limits the ability to draw firm conclusions regarding optimal management strategies.

Despite these limitations, this narrative approach allows a comprehensive and clinically oriented synthesis of current evidence, highlighting consistent patterns of risk and identifying key areas where further prospective and standardized research is needed.

## 9 Conclusions

Childhood obesity is a highly prevalent chronic condition and an important modifier of surgical risk. Children with obesity undergoing abdominal surgery may experience higher rates of perioperative respiratory events, wound complications, longer operative times, and prolonged hospital stay, particularly in the presence of comorbidities such as obstructive sleep apnea syndrome and cardiometabolic dysfunction. Obesity should therefore be considered a risk amplifier rather than a contraindication to surgery, as acceptable safety profiles can be achieved in appropriately equipped centers with experienced multidisciplinary teams. Effective management requires systematic preoperative identification of obesity and associated comorbidities, tailored anesthetic and surgical planning, and vigilant postoperative care. Structured perioperative pathways and organizational preparedness are important to identify patients requiring higher-intensity monitoring. Multidisciplinary strategies integrated within ERAS pathways adapted to pediatric obesity may represent a promising approach to optimize perioperative management and resource utilization in this growing patient population. However, further prospective studies are needed to standardize obesity definitions and to better characterize long-term patient-centered outcomes, including quality of life and functional recovery.
