# Supplementary material for: Impact of pediatric obesity on abdominal surgery outcome: a narrative review
Source: Front Pediatr. 2026 Mar 25;14:1780811. doi: 10.3389/fped.2026.1780811 (PMC13057296; doi:10.3389/fped.2026.1780811)
Supplement: Supplementary file 1 [file Datasheet1.pdf]

Table S1. Literature search strings used for each database (PubMed, Embase, and Scopus).

PubMed	Embase	Scopus
(obesity OR “pediatric obesity”) AND (child OR children OR adolescent) AND (“abdominal surgery” OR “general surgery”) AND (“perioperative complications” OR “postoperative complications” OR anesthesia OR “wound healing” OR “obstructive sleep apnea” OR “cardiovascular disease” OR inflammation)	('obesity'/exp OR 'pediatric obesity' OR obesity) AND ('child'/exp OR child OR adolescent) AND ('abdominal surgery'/exp OR 'general surgery') AND ('perioperative complication'/exp OR 'postoperative complication'/exp OR anesthesia OR 'wound healing' OR 'obstructive sleep apnea' OR 'cardiovascular disease' OR inflammation)	TITLE-ABS-KEY (obesity OR “pediatric obesity”) AND TITLE-ABS-KEY (child OR adolescent) AND TITLE-ABS-KEY (“abdominal surgery” OR “general surgery”) AND TITLE-ABS-KEY (“perioperative complications” OR “postoperative complications” OR anesthesia OR “wound healing” OR “obstructive sleep apnea” OR “cardiovascular disease” OR inflammation)
